# Supplementary material for: Single-pixel p-graded-n junction spectrometers
Source: Nat Commun. 2024 Feb 27;15:1773. doi: 10.1038/s41467-024-46066-5 (PMC10899627; doi:10.1038/s41467-024-46066-5)
Supplement: Supplementary file 1 — Supplementary Information [file 41467_2024_46066_MOESM1_ESM.pdf]

Supplementary Information of Single-pixel p-graded-n Junction Spectrometers

Jingyi Wang[1,3†], Beibei Pan[1,3†], Zi Wang[1,2,4†], Jiakai Zhang[1], Zhiqi Zhou[1,3], Lu Yao[1,3], Yanan Wu[1], Wuwei Ren[1], Jianyu Wang[1,2,4], Haiming Ji[4], Jingyi Yu[1*], Baile Chen[1,3*]

[1]School of Information Science and Technology, ShanghaiTech University, Shanghai, 201210, P. R. China.

[2]Shanghai Institute of Technical Physics, Chinese Academy of Sciences, Shanghai, 200083, P. R. China.

[3] Shanghai Engineering Research Center of Energy Efficient and Custom AI IC, Shanghai, 201210, P. R. China.

[4]University of Chinese Academy of Sciences, Beijing, 100049, P. R. China.

*Corresponding author(s). E-mail(s): yujingyi@shanghaitech.edu.cn; chenbl@shanghaitech.edu.cn;

Contributing authors: wangjy8@shanghaitech.edu.cn; panbb@shanghaitech.edu.cn; wangzi@shanghaitech.edu.cn; zhangjk@shanghaitech.edu.cn; zhouzq@shanghaitech.edu.cn; yaolu@shanghaitech.edu.cn; wuyn1@shanghaitech.edu.cn; renww@shanghaitech.edu.cn; jywang@mail.sitp.ac.cn; jhm@semi.ac.cn;

†These authors contributed equally to this work.

1 Working mechanism of p-graded-n junction spectrometer

In a traditional pn junction photodiode, as the incident photons get absorbed within the device, the photo-generated electron-hole pairs will be collected by diffusion or drift under the electrical field across the depletion region. As the reverse bias increases, the carrier collection efficiency will increase, as more carriers are collected before recombined, resulting in an improved quantum efficiency or responsivity. However, the responsivity versus wavelength curves at different reverse biases are highly correlated. Therefore, the traditional pn junction photodiodes can not be used for computational spectrometers.

In our implementation, we design a p-graded-n junction, where the n-doped material uses the compositionally graded $Al_xGa_{1-x}As$, with x grading from 0.5 to 0. Therefore, this gradient n-doped material has higher bandgap AlGaAs near the junction, and narrower bandgap AlGaAs or GaAs far away from the junction. When the reverse bias is small, only the large bandgap AlGaAs is depleted. Therefore, only the carriers generated by the shorter wavelength incident light, which is absorbed in the large bandgap AlGaAs layer, can be collected efficiently and contribute to the photocurrent. The holes generated in the narrow bandgap AlGaAs or GaAs layer, created by the longer wavelength incident light, are blocked by the valence band barrier of large bandgap AlGaAs, and therefore, can not contribute photocurrent efficiently. As the reverse bias increases, the depletion region extends into the narrower bandgap AlGaAs. These carriers generated by the longer wavelength incident light can then be collected and contribute photocurrent. Therefore, we can obtain a voltage-tunable-spectrum responsivity, which is critical for latter spectrum reconstruction.

The advantages of this p-graded-n junction spectrometer are numerous. Firstly, it can achieve high-resolution wavelength spectrometry with a single-pixel device, eliminating the need for multiple discrete detectors or gratings. Secondly, this structure can be implemented with other material systems with different bandgaps, which could be used for spectrometer application in other wavelength bands, such as near-infrared or mid-wavelength infrared bands. Thirdly, the device architecture can be employed in the Focal Plane Array (FPA) with flip-chip bond technology, by inverting the whole epi-stack for back-illumination. The compatibility with FPA suggests that this p-graded-n junction spectrometer can be integrated into large-scale FPAs, allowing for spectral imaging applications. The schematic picture of the FPA using single-pixel photodetectors can be found in the section "Scalability and reproducibility".

2 Bandgap and composition relation of $Al_xGa_{1-x}As$ material

The relation between bandgap and composition of $Al_xGa_{1-x}As$ material with an x range from 0 to 0.5 could be described with the equation below[1]:

$$E_g = 1.247x + 1.424eV \tag{1}$$

where x is the Al composition. It can be concluded from the above formula that the band gap of the material changes linearly with the gradual change of Al components.

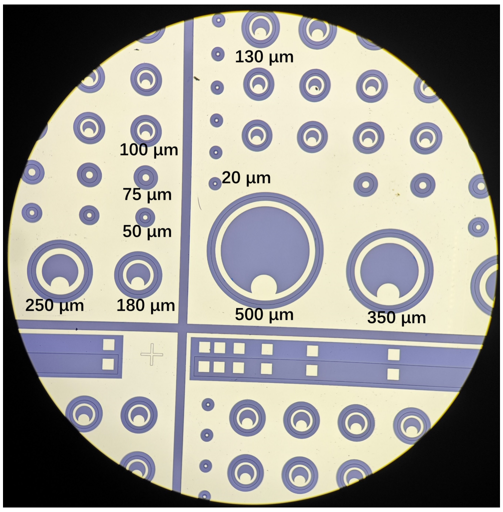

Supplementary Fig. 1 Microscope image of the p-graded-n devices with different footprint.

3 Footprint of the p-graded-n design

The Supplementary Fig. 1 shown below is the microscope image of the fabricated p-graded-n spectrometers with different mesa diameters ranging from 20 μm to 500 μm. To couple the incident light into the device active region efficiently, we selected 180 μm diameter devices for measurement.

The functionality of the p-graded-n spectrometer is independent of the size of the device, as long as the incident light is shining from the top of the mesa. We believe it may not gain additional benefit by shrinking the device diameter below μm range, since it is difficult for optical coupling, and the alignment tolerance would be challenging.

4 Scalability and reproducibility

Our p-graded-n junction is based on the III-V semiconductor materials, and can be fabricated on 3-inch GaAs wafers in III-V compatible foundries. For hyperspectral imaging applications, the p-graded-n devices array can be flip-chip bonded to a readout integrated circuit (Readout IC) with indium bumps, as shown in

Supplementary Fig. 2. For back-illumination in the FPA applications, the epi-layer stacks should be inverted with the GaAs substrate removed.

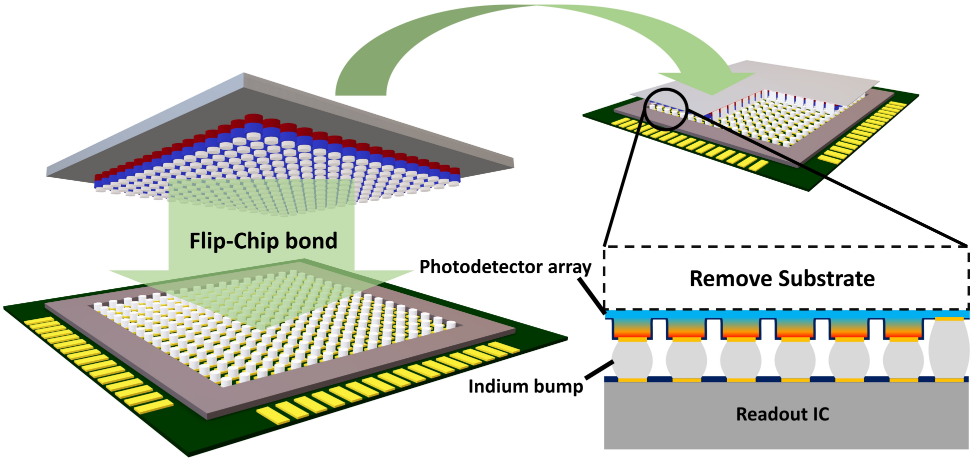

Supplementary Fig. 2 Schematic diagram of multiple single pixel p-graded-n spectrometer arrays.

To evaluate the device-to-device variation, we measured the responsivity matrix of two different devices (Device A and Device B). The comparison of the two devices is shown in Supplementary Fig. 3. The figure shows the responsivity under 5 bias voltages of -2, -4, -6, -8 and -10 V. The mean difference between the two devices is 1.01%, which indicates good uniformity.

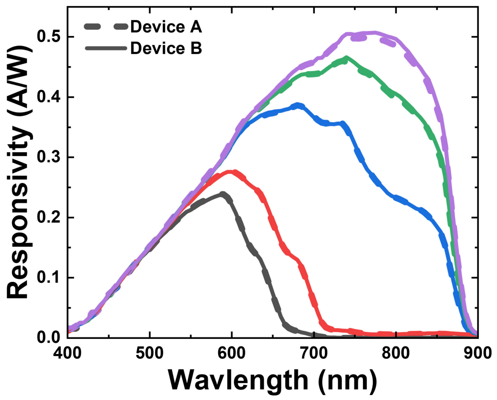

Supplementary Fig. 3 Comparison of the responsivity of two different devices (Device A and Device B).

5 Fabrication flow

The epitaxial layer structure of the detector is grown with Metal-organic Chemical Vapor Deposition (MOVCD). The fabrication process has three main steps. First, the photoresist (AZ5214) was spun on the grown epitaxial structure for lithography. Through the first step of lithography, wet etching with $H_3PO_4 : H_2O_2 : H_2O$ solution (1: 1: 10)is used to form device mesa. Next, a second photolithography step is performed for the p and n metal contact. Both p and n metal contact use Ti/Pt/Au, which is deposited with Denton EXPLOER-14 E-beam system.

Silicon dioxide passivation layer is then deposited on the device sidewall and surface with Plasma Enhanced Chemical Vapor Deposition (PECVD), this layer also serve as anti-reflecting coating, which enhances the device responsivity.

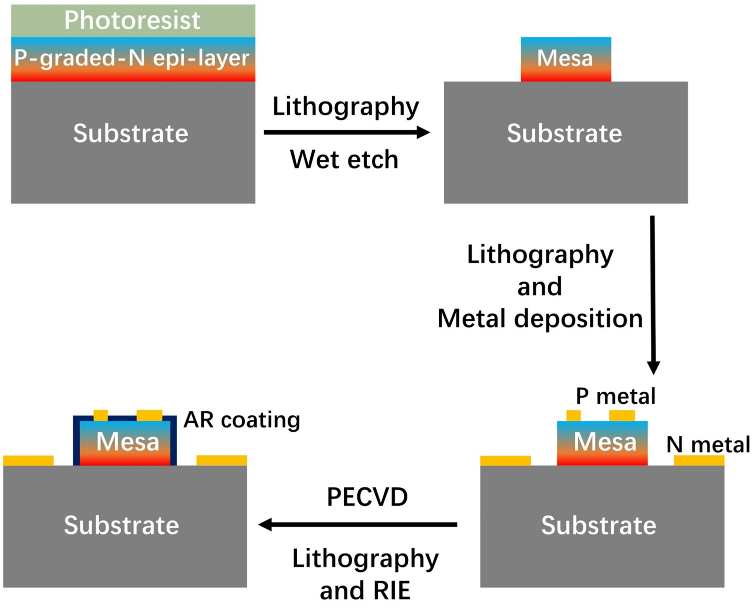

Supplementary Fig. 4 Fabrication flow of the p-graded-n spectrometers

6 Power linearity of the device

The power linearity of the p-graded-n spectrometer with 180 μm diameter was measured with a 532 nm laser. A beam splitter is used to separate the light into two paths. One path of the laser is shining on the device, while the other path is used to calibrate the power of the laser. The power of the laser beam can be varied with the optical attenuator. The measurement setup is shown in Supplementary Fig. 5 a.

The measurement results are illustrated in Supplementary Fig. 5 b. The device demonstrates a good linearity within the power range from 50 nW to 75 mW. For the optical power below 20 nW, the incident optical power can not be accurately calibrated, limited by the sensitivity of the optical power meter. Given the diameter of the focused laser spot of about 50 microns, it corresponds to an optical power density range of 5×10^{-3} W/cm^2 to 3.75×10^3 W/cm^2.

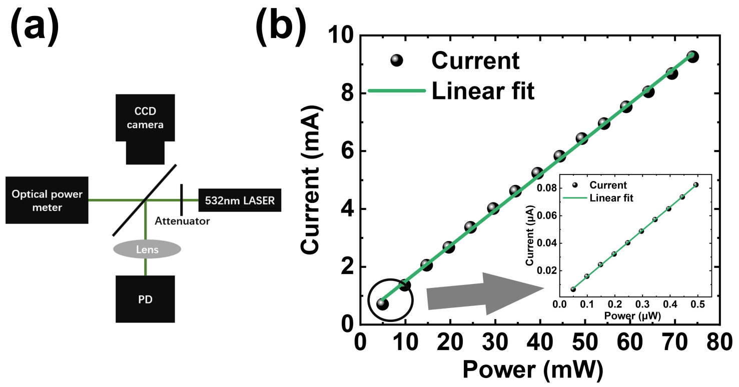

Supplementary Fig. 5 a. Power linearity measurement setup and b. the power linearity of the device

7 Comparison with recent works

In Supplementary Table 1, we present a comprehensive comparison between our work and recent studies, covering key aspects such as principle, footprint, resolution, wavelength accuracy, spectral range, dark current density, and responsivity.

Additionally, we compare dark current density and responsivity with other photodetector-based compact spectrometers. Our p-graded-n junction exhibits superior single-device performance, showcasing both low dark current and high responsivity when compared to other photodetector-based compact spectrometers.

The p-graded-n spectrometer utilizes a single detector with a variable footprint down to 10 microns, achieving a resolution of 10 nm and a wavelength accuracy of 0.30 nm. These specifications are comparable to those reported in prior works. Notably, the footprint of our device is independent of its size, providing flexibility for adjustment—making it suitable for ultra-compact designs or larger footprints for enhanced coupling efficiency with different lithography patterns.

Data for the comparison are primarily sourced from the referenced articles and their supplementary materials. Part of the dark current and responsivity data was extracted from the figures in the respective references, with dark current density calculations based on the claimed device footprint.

Type	Principle	Type	Footprint	Resolution	Wavelength accuracy	Spectral Range	Dark current density	Responsivity
Computational	P-graded-n junction(this work)	Single detector	Various radius down to 10 μm	10 nm	0.30 nm	480-820 nm	$3.47 \times 10^{-9} A/cm^2$	0.51 A/W
Computational	Van der Waal heterojunction[2]	Single detector	$22 \times 8\ \mu m^2$	3 nm	0.36 nm	405-845 nm	$1 \times 10^{-3} A/cm^2$	1.2×10^{-3} A/W
Computational	Van der Waal heterostructure[3]	Single detector	$6 \times 4\ \mu m^2$	20 nm	N/A	1150-1470 nm	$4.17 \times 10^{-5} A/cm^2$	1×10^{-5} A/W
Computational	Black phosphorus[4]	Single detector	$16 \times 9\ \mu m^2$	90 nm	N/A	2000-9000 nm	N/A	N/A
Computational	Single anowire[5]	Detector arrays	$75 \times 0.5\ \mu m^2$	10 nm	N/A	500-630 nm	0.27 A/cm^2	10^4 A/W
Computational	Structurally colored silicon nanowires[6]	Detector arrays	2×2 mm^2	6 nm	N/A	450-800 nm	$2.92 \times 10^{-6} A/cm^2$	0.25 A/W
Computational	Fishnet structure[7]	Detector arrays	$200 \times 200\ \mu m^2$	16.5 nm	N/A	400-850 nm	$1.25 \times 10^{-5} A/cm^2$	8.8×10^{-2} A/W
Computational	Photonic crystal slabs[8]	Filter arrays	$210 \times 210\ \mu m^2$	1.5 nm	N/A	550-750 nm	N/A	N/A
Conventional	Dielectric metasurfaces[9]	Filter arrays	1.5×1.5 mm^2	16 nm	N/A	3600-4600 nm	N/A	N/A
Computational	Mach-Zehnder interferometer[10]	Filter arrays	2.8×0.6 mm^2	0.2 nm	N/A	1550-1570 nm	N/A	N/A
Computational	Colloidal quantum dots[11]	Filter arrays	8.5×6.8 mm^2	2 nm	N/A	390-690 nm	N/A	N/A
Computational	Dielectric metasurfaces[12]	Filter arrays	5.6×5.6 mm^2	1.2 nm	N/A	450-780 nm	N/A	N/A
Conventional	Dielectric metasurfaces[13]	Filter arrays	2×2 mm^2	1 nm	N/A	820-890 nm	N/A	N/A

Supplementary Table 1 Comparison of different miniaturized spectrometers.

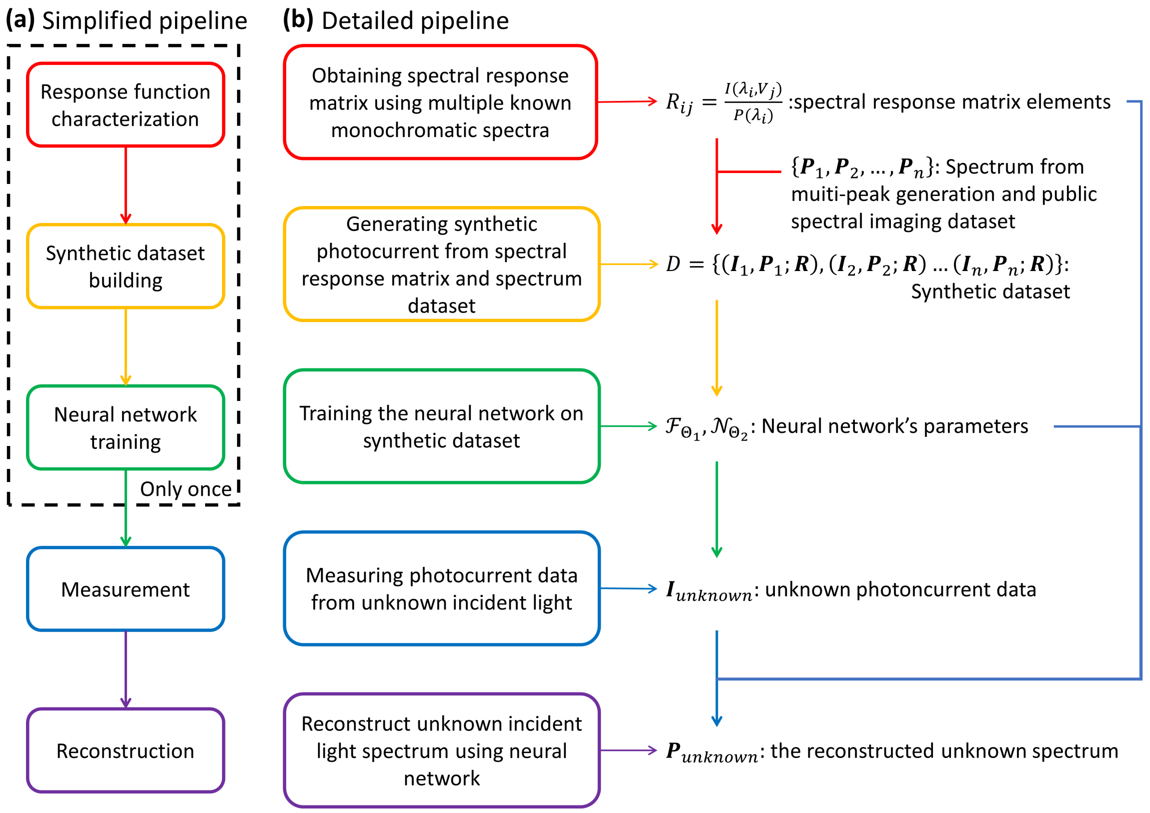

Supplementary Fig. 6 Pipeline diagram. a. Simplified pipeline. **b.** Detailed pipeline. Based on the characterized spectral response function, a synthetic dataset is built to train the neural network. The well-trained neural network is able to reconstruct the spectra from the measured photocurrents of our spectrometers.

8 Reconstruction problem statement

In the context of utilizing our spectrometer for the measurement of an unfamiliar spectrum, the photocurrent, denoted as I and measured at a bias voltage V, can be formulated as an integration equation involving the unknown spectrum $\boldsymbol{P}(\lambda)$ and the pre-calibrated spectral responsivity $\boldsymbol{R}(\lambda, V)$:

$$\boldsymbol{I}(V) = \int_{\lambda_{min}}^{\lambda_{max}} \boldsymbol{R}(\lambda, V)\boldsymbol{P}(\lambda)d\lambda \tag{2}$$

where $\boldsymbol{I}(V)$ is the measured photocurrent curve by our spectrometer and the integration range $(\lambda_{min}, \lambda_{max})$ denote the minimum and maximum functional wavelengths of the spectrometer, respectively.

In the deployment of the spectrometer, the bias voltage (V) applied to the device manifests as a discrete sequence of length M, denoted as $\{V_1, V_2, ..., V_M\}$. The corresponding photocurrents $(I(V_i))$, where $i = 1, 2, ..., M$, is determined at each bias voltage. Let I_i be defined as $I_i = I(V_i)$. Simultaneously, the incident spectrum $(\boldsymbol{P})$ is represented as a discrete sequence of length N, denoted as $\{P_{\lambda_1}, P_{\lambda_2}, ..., P_{\lambda_N}\}$, with the corresponding spectral values $P(\lambda_i)$. Equation (2) can then be discretized as follows:

$$\begin{bmatrix} I_1 \\ I_2 \\ \vdots \\ I_M \end{bmatrix} = \begin{bmatrix} R_{1,1} & R_{1,2} & \cdots & R_{1,N} \\ R_{2,1} & R_{2,2} & \cdots & R_{2,N} \\ \vdots & \vdots & \ddots & \vdots \\ R_{M,1} & R_{M,2} & \cdots & R_{M,N} \end{bmatrix} \begin{bmatrix} P_1 \\ P_2 \\ \vdots \\ P_N \end{bmatrix} \tag{3}$$

it can be simplified as:

$$\boldsymbol{I} = \boldsymbol{R}\boldsymbol{P} \tag{4}$$

Numerous classical optimization-based numerical methods, including Mean Squared Error (MSE), Tikhonov regression, and sparse L1 regularization, can be employed to deduce unknown spectra. However, these methods may not adeptly exploit the distinctive characteristic of 'voltage accumulative' inherent in our device (refer to section 12 for experimental validation). Consequently, a tailored approach for our device involves the implementation of the Neural Spectral Fields algorithm.

9 Response function characterization

In subsequent stages of the pipeline, spectral response functions R_{ij} are indispensable and therefore require pre-calibration. The calibration process employs a monochromator to generate quasi-monochromatic light with a bandpass of approximately 2.31 nm. This light is directed onto the device through the lens and optical fibers, resulting in photoelectric current generation. The incident light spans wavelengths from 400 nm to 900 nm, with the monochromator's optical power normalized against a standard responsivity detector. The device is subjected to biases ranging from 0 V to -10 V. The response matrix measurement setup can be found

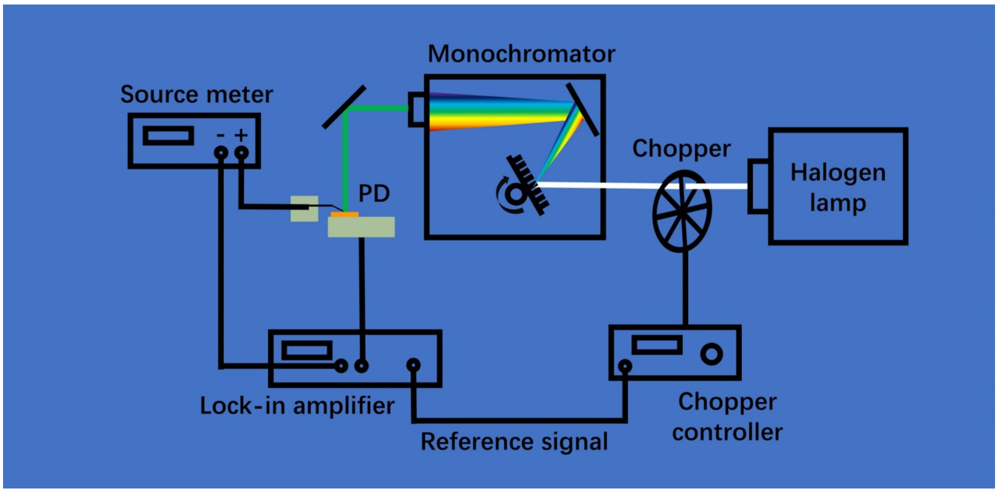

Supplementary Fig. 7 Responsivity measurement setup sketch

in Supplementary Fig. 7. To accurately measure the response matrix, the incident light signal is modulated by a 180 Hz chopper and the reference frequency is sent to an lock-in amplifier. The photodetector is biased with a source meter, a halogen lamp provides broadband light to the monochromator.

Each element of the spectral response functions, denoted as R_{ij}, is defined as $R_{ij} = \frac{I(\lambda_i,V_j)}{P(\lambda_i)}$. Here, $I(\lambda_i, V_j)$ represents the response current of the device at wavelength λ_i and bias V_j, normalized by the intensity of the incident light $P(\lambda_i)$. The calibrated spectral response functions are stored as a matrix and play a pivotal role in subsequent stages of the pipeline.

10 Synthetic dataset construction

When employing our method to reconstruct spectra, it is imperative to pre-construct a synthetic dataset for training the neural network. The synthetic dataset primarily comprises spectra $\boldsymbol{P}$, corresponding currents $\boldsymbol{I}$, and response functions $\boldsymbol{R}$. The spectra in the synthetic dataset originate from two sources. For multiple peaks reconstruction, we generate Gaussian multi-peak functions to simulate spectra produced by lasers:

$$\boldsymbol{P} = \sum_{i=1}^{2} A_i \exp\{-\frac{(\lambda - \lambda_i)}{2\sigma_i^2}\} \tag{5}$$

Here, A_i represents the height of each peak, λ_i is the central wavelength of each peak, and σ_i is related to the width of the peak. For single-peak data generation, we set $A_1 = 1.0, A_2 = 0.0$, λ_i is uniformly distributed random numbers in the range $[480\,\text{nm}, 820\,\text{nm}]$, and σ_i ensures the generated peak widths are uniformly

distributed random numbers in the range $[5\,\text{nm}, 100\,\text{nm}]$. For dual-peak data generation, we set A_1 and A_2 as uniformly distributed random numbers in the range $[0.8, 1.2]$, λ_i is uniformly distributed random numbers in the range $[480\,\text{nm}, 820\,\text{nm}]$, and σ_i ensures the generated peak widths are uniformly distributed random numbers in the range $[5\,\text{nm}, 20\,\text{nm}]$. More complex multi-peak functions, such as the reconstruction of a three-peak function (see Section 14), can also be added to the simulated dataset to enable accurate reconstruction of intricate spectra.

The second source of spectra in the synthetic dataset is the natural light spectrum. We selected all laboratory scene data from the ICVL(Imperial Computer Vision & Learning) dataset [14] containing calibration boards. The spectral images were calibrated using the spectrum of the calibrated whiteboard in the scene. The calibrated spectrum for each pixel was then added to the dataset.

Once the spectral data is prepared, we compute the corresponding currents $\boldsymbol{I}$ using the calibrated response function $\boldsymbol{R}$ (see Section 9) and the discretized model of the spectrometer (see Eqn. 4). Additionally, a Gaussian noise of 0.1% is added to the computed currents to simulate measurement errors.

On the constructed dataset $D = \{(\boldsymbol{I}_1, \boldsymbol{P}_1; \boldsymbol{R}), (\boldsymbol{I}_2, \boldsymbol{P}_2; \boldsymbol{R}), \cdots, (\boldsymbol{I}_n, \boldsymbol{P}_n; \boldsymbol{R})\}$, we train Neural network for additional spectrum reconstruction.

11 Neural network training

Our reconstruction algorithm comprises two neural networks: the Feature Extractor (FE) and the Neural Spectral Fields (NSF). We denote the Feature Extractor as $\mathcal{F}_{\Theta_1}$, where Θ_1 represents its parameters, and the Neural Spectral Fields as $\mathcal{N}_{\Theta_2}$, with Θ_2 denoting its parameters.

Firstly, we need to train the Feature Extractor on the constructed dataset to enable it to extract deep features from the measured current-voltage curves. During training, the input consists of current-voltage curves, and we aim for the Feature Extractor to directly output features. However, as our dataset lacks explicit features, we cannot directly utilize the output of the Feature Extractor for training. Consequently, we simultaneously train the Feature Extractor and Neural Spectral Fields using the spectra from the dataset as output:

$$\min_{\Theta_1, \Theta_2} \sum_{i=1}^{n} \|\mathcal{N}_{\Theta_2}(\mathcal{F}_{\Theta_1}(\boldsymbol{I}_i)) - \boldsymbol{P}_i\|_2^2 \tag{6}$$

During network training, we executed 100,000 epochs with a batch size of 1,000. The learning rate was set to 0.001, and for every 20,000 epochs, the learning rate was multiplied by 0.8. We employed the Adam optimizer with beta parameters set to 0.9 and 0.999, and epsilon set to 1×10^{-8}. The training duration for our neural network spans approximately 3 hours, conducted on a NVIDIA GeForce GTX 1050 Ti.

Upon completing training, for a given device photocurrent $\boldsymbol{I}$, the neural networks can provide the predicted incident light spectrum $\widehat{\boldsymbol{P}}$:

$$\widehat{\boldsymbol{P}} = \mathcal{N}_{\Theta_2}(\mathcal{F}_{\Theta_1}(\boldsymbol{I}_i)) \tag{7}$$

The prediction time of our neural network is nearly instantaneous, requiring only 0.84 seconds for each individual sample. Moreover, the neural network exhibits commendable parallel performance, consuming a total of 1.04 seconds for the prediction of 10,000 measured I-V curves. The execution of runtime tests is conducted on a NVIDIA GeForce GTX 1050 Ti.

12 Effects of measurement errors

To rigorously evaluate the robustness of different methods against noise in the testing phase, we generated simulated data at varying noise levels. Gaussian noise, characterized by a zero mean and variations of 0.1%, 1%, 10%, 20%, 30%, 50%, 80%, and 100% of the maximum value of the measured I-V curve, was introduced. The application of noise in this context pertains to the measured I-V curve. Subsequently, we conducted tests on the simulated data, employing both the neural network reconstruction method and the Tikhonov

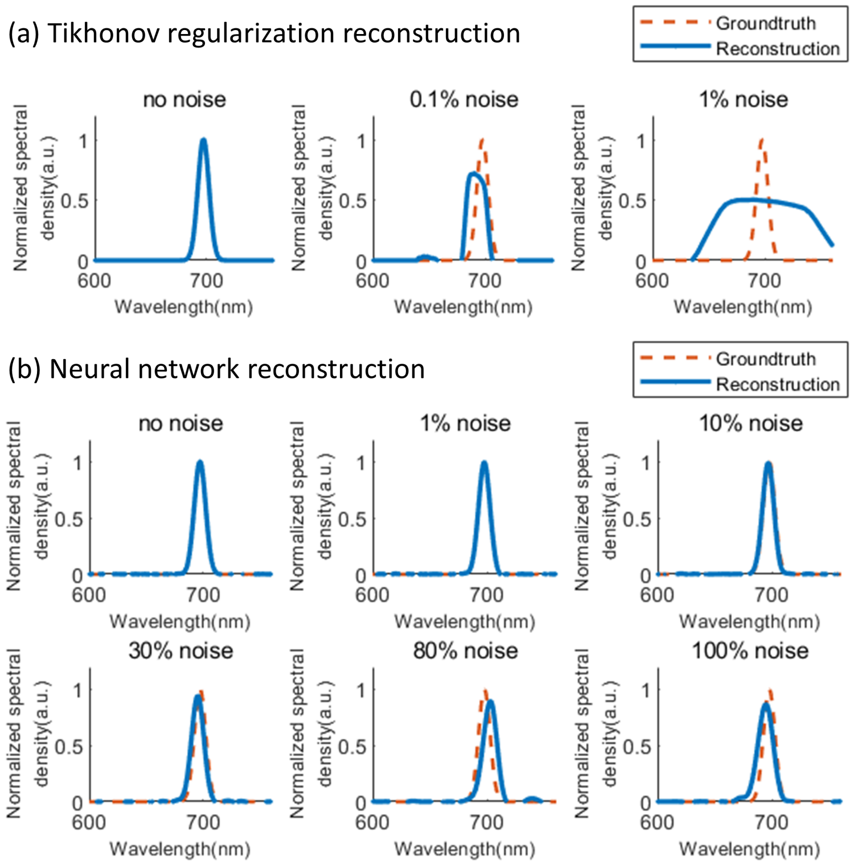

Supplementary Fig. 8 Effects of measurement errors. a. Reconstruction results employing Tikhonov regularization under various noise levels. **b.** Reconstruction results Using the proposed neural network method under different levels of noise. The proposed neural network method exhibits superior noise robustness compared to Tikhonov regularization when applied to simulated data. Even under 30% simulated noise, the neural network method accurately reconstructs spectra. Remarkably, even under 100% simulated noise conditions, the peak wavelength error in the neural network reconstruction does not exceed 10nm.

regularization reconstruction method. Supplementary Fig. 8 illustrates the outcomes, demonstrating that the neural network method adeptly reconstructs incident spectra even under the influence of 30% intense noise.

13 Broadband reconstruction

We have examined the reconstruction error, as depicted in Supplementary Fig. 9. Our evaluation of the spectrum reconstruction performance in relation to the peak width reveals that the reconstruction error increases as the peak width expands. Supplementary Fig. 9(a) and (b) show the reconstructed I-V curve derived from algorithmic prediction, while the ideal I-V curve is the product of the response function and the ground-truth spectrum. It should be noted that the ideal I-V curve can be considered as reference.

The accuracy of the reconstruction in terms of peak width should be considered from two perspectives: unreal synthetic data and algorithm reconstruction error. Firstly, the measured I-V curve doesn't perfectly represent the product of the response function and the spectrum. This discrepancy could be due to the inherent response function of the ground truth from built-in detector in the commercial spectrometer. Consequently, the synthetic data for training is biased and lacks an exact distribution from the real experimental data. As illustrated in Supplementary Fig. 9 (a) and (b), the real I-V curve diverges from the ideal one, and the reconstruction error escalates with the increase of difference between real I-V curve and reconstructed one.

Second, Supplementary Fig. 9 (c) exhibits slight distortion as the peak width increases, which can be attributed to the laser source used in the experiments. Given that the single peak spectrum is primarily modeled as a Gaussian function in synthetic data construction (please refer to the "Synthetic dataset

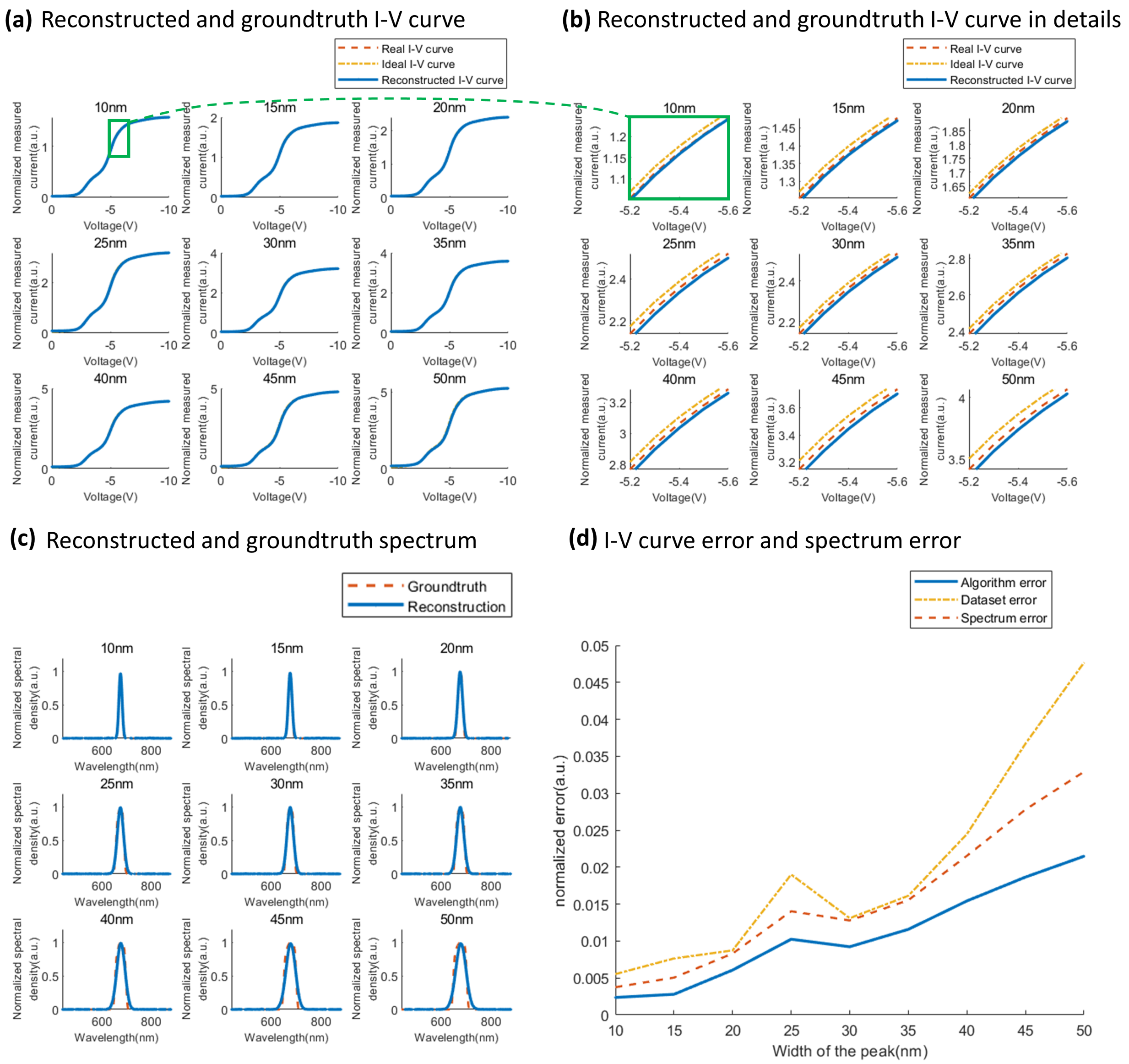

Supplementary Fig. 9 Analysis of broadband reconstruction. a,b. The comparison of real I-V curve, ideal I-V curve and reconstructed I-V curve. **c.** The reconstructed spectrum and ground-truth regarding to the width of the peak from 10nm to 50nm. **d.** The trend of spectrum error, algorithm error and dataset error regarding to the peak width.

construction" section in the supplementary material for details), it is reasonable that the reconstructed I-V curve deviates from the actual one. Supplementary Fig. 9 (d) provides a more detailed analysis of the relationship between different errors. The dataset error is defined as the Mean Average Error (MAE) between the real and ideal I-V curve. The spectrum error is defined as the MAE between the reconstructed spectrum and the ground truth. The algorithm error is defined as the MAE between the reconstructed and real I-V curve. It is evident that the spectrum error increases as both the dataset error and algorithm error increase.

14 Multi-peak reconstructions

In order to further assess the effectiveness of our method on more complicated spectra, we employed our device to detect incident light with three and four peaks. We first train the neural network using only three-peak and four-peak synthetic data respectively.

Supplementary Fig. 10(a) and (c) display the reconstructed spectrum and I-V curve of three-peak reconstruction tasks. Supplementary Fig. 10(a) reveals that our neural network method effectively reconstructs the three-peak spectrum, albeit with some existing artifacts. Supplementary Fig. 10(c) further demonstrates that the reconstructed I-V curve aligns closely with the real I-V curve, suggesting that the algorithm has

accurately learned the physical constraints of the spectrometer model. However, the real I-V curve deviates from the ideal one, indicating that the synthetic dataset used for training differs from the real ground-truth experimental data, leading to artifacts and errors in the reconstructed spectrum as shown in Supplementary Fig. 10(a). The discrepancy between the real and ideal I-V curve could arise from the intrinsic response function of the commercial spectrometer used to measure the ground truth spectrum.

Supplementary Fig. 10(b) and (d) present the reconstructed spectrum and I-V curve of four-peak reconstruction tasks. Supplementary Fig. 10(b) shows that our neural network method can reconstruct the main peak of the four-peak spectrum. However, the reconstruction of the four-peak spectrum is not as precise as that of the three-peak spectrum. Supplementary Fig. 10(d) provides an explanation for this. It shows that the reconstructed I-V curve derived from the algorithm does not fit the real I-V curve well. This could be attributed to the higher complexity of the four-peak spectrum compared to the three-peak spectrum. Given that the proposed physics-based refinement only applies simple operations such as shift and scale, the reconstructed spectrum, with its artifacts, does not perfectly fit the real I-V curve.

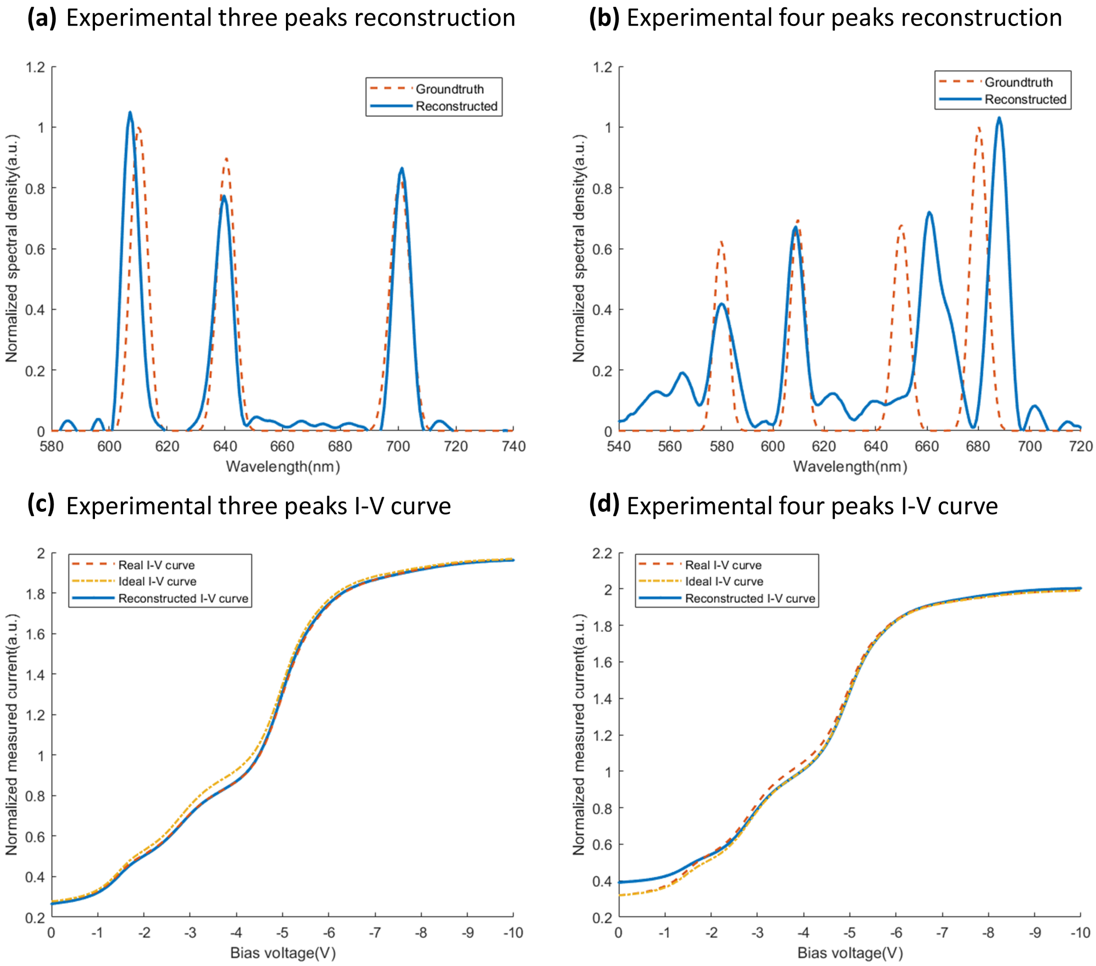

Supplementary Fig. 10 Results of multipeaks. a. The center wavelengths of the experimental three-peak spectrum are 610 nm, 640 nm and 700 nm respectively. The corresponding reconstructed center wavelengths are 607 nm, 640 nm and 701 nm. **b.** The center wavelengths of the experimental four-peak spectrum are 580 nm, 610 nm, 650 nm, and 680 nm, respectively. The corresponding reconstructed center wavelength are 580 nm, 609 nm, 661 nm, and 688 nm. **c.** In the three-peak Reconstruction, the reconstructed I-V curve aligns closely with the actual I-V curve. However, the real I-V curve deviates from the ideal one. **d.** In the four-peak Reconstruction, the reconstructed I-V curve derived from the algorithm does not fit the real I-V curve well.

References

[1] Adachi, S.: GaAs and Related Materials, 2nd edn. WORLD SCIENTIFIC, P O Box, 128 Farrer Road, Singapore, 912805 (1994). https://doi.org/10.1142/2508

[2] Yoon, H.H., Fernandez, H.A., Nigmatulin, F., Cai, W., Yang, Z., Cui, H., Ahmed, F., Cui, X., Uddin, M.G., Minot, E.D., Lipsanen, H., Kim, K., Hakonen, P., Hasan, T., Sun, Z.: Miniaturized spectrometers with a tunable van der waals junction. Science **378**(6617), 296–299 (2022) https://doi.org/10.1126/science.add8544 https://www.science.org/doi/pdf/10.1126/science.add8544

[3] Deng, W., Zheng, Z., Li, J., Zhou, R., Chen, X., Zhang, D., Lu, Y., Wang, C., You, C., Li, S., Sun, L., Wu, Y., Li, X., An, B., Liu, Z., Wang, Q.j., Duan, X., Zhang, Y.: Electrically tunable two-dimensional heterojunctions for miniaturized near-infrared spectrometers. Nature Communications **13**(1), 4627 (2022) https://doi.org/10.1038/s41467-022-32306-z

[4] Yuan, S., Naveh, D., Watanabe, K., Taniguchi, T., Xia, F.: A wavelength-scale black phosphorus spectrometer. Nature Photonics **15**(8), 601–607 (2021) https://doi.org/10.1038/s41566-021-00787-x

[5] Yang, Z., Albrow-Owen, T., Cui, H., Alexander-Webber, J., Gu, F., Wang, X., Wu, T.-C., Zhuge, M., Williams, C., Wang, P., Zayats, A.V., Cai, W., Dai, L., Hofmann, S., Overend, M., Tong, L., Yang, Q., Sun, Z., Hasan, T.: Single-nanowire spectrometers. Science **365**(6457), 1017–1020 (2019) https://doi.org/10.1126/science.aax8814 https://www.science.org/doi/pdf/10.1126/science.aax8814

[6] Meng, J., Cadusch, J.J., Crozier, K.B.: Detector-only spectrometer based on structurally colored silicon nanowires and a reconstruction algorithm. Nano Letters **20**(1), 320–328 (2020) https://doi.org/10.1021/acs.nanolett.9b03862

[7] Cadusch, J.J., Meng, J., Craig, B., Crozier, K.B.: Silicon microspectrometer chip based on nanostructured fishnet photodetectors with tailored responsivities and machine learning. Optica **6**(9), 1171–1177 (2019) https://doi.org/10.1364/OPTICA.6.001171

[8] Wang, Z., Yi, S., Chen, A., Zhou, M., Luk, T.S., James, A., Nogan, J., Ross, W., Joe, G., Shahsafi, A., Wang, K.X., Kats, M.A., Yu, Z.: Single-shot on-chip spectral sensors based on photonic crystal slabs. Nature Communications **10**(1), 1020 (2019) https://doi.org/10.1038/s41467-019-08994-5

[9] Tittl, A., Leitis, A., Liu, M., Yesilkoy, F., Choi, D.-Y., Neshev, D.N., Kivshar, Y.S., Altug, H.: Imaging-based molecular barcoding with pixelated dielectric metasurfaces. Science **360**(6393), 1105–1109 (2018)

[10] Kita, D.M., Miranda, B., Favela, D., Bono, D., Michon, J., Lin, H., Gu, T., Hu, J.: High-performance and scalable on-chip digital fourier transform spectroscopy. Nature Communications **9**(1), 4405 (2018) https://doi.org/10.1038/s41467-018-06773-2

[11] Bao, J., Bawendi, M.G.: A colloidal quantum dot spectrometer. Nature **523**(7558), 67–70 (2015) https://doi.org/10.1038/nature14576

[12] Faraji-Dana, M., Arbabi, E., Arbabi, A., Kamali, S.M., Kwon, H., Faraon, A.: Compact folded metasurface spectrometer. Nature communications **9**(1), 4196 (2018)

[13] Yesilkoy, F., Arvelo, E.R., Jahani, Y., Liu, M., Tittl, A., Cevher, V., Kivshar, Y., Altug, H.: Ultrasensitive hyperspectral imaging and biodetection enabled by dielectric metasurfaces. Nature Photonics **13**(6), 390–396 (2019)

[14] Arad, B., Ben-Shahar, O.: Sparse recovery of hyperspectral signal from natural rgb images. In: Computer Vision–ECCV 2016: 14th European Conference, Amsterdam, The Netherlands, October 11–14, 2016, Proceedings, Part VII 14, pp. 19–34 (2016). Springer
